# The rise of ACS and its importance

**DOI:** 10.1186/s13017-024-00538-7

**Published:** 2024-03-08

**Authors:** Brian WCA Tian

**Affiliations:** Department of General Surgery, Singapore General Hospital, Outram Road, Singapore, S169608 Singapore

**Keywords:** Acute care, Trauma, Health systems

## Introduction

Acute care surgery [ACS] as a model of care and a focused area of specialisation is gaining traction globally [1–3]. ACS is seen as a natural evolution of the specialty of trauma. If anything, this restructuring is desperately needed.

In the ideal ACS system, I propose that surgeons will be:

1. Exposed to a wide variety of operative procedures and techniques, including the latest laparoscopic and robotic skill sets .
2. The trauma surgeon will get a high operative load weekly, if not daily, to remain fresh and sharp.
3. The ACS surgeon should constantly advance research and development in emergency work, which is often neglected [4].

Globally, every country runs its own version of the ACS model. Despite the variation in systems, the ACS model has generally been shown to reduce time to surgery and complication rates, particularly for common conditions such as appendicitis and cholecystitis [5–7]. The productivity of the department as a whole also improves, with greater utilisation of the theatre and intensive care unit (ICU) [8]. Some studies reported reductions in length of stay (LOS), complications and costs compared to those in standard care units [9, 10]. Apart from patient driven outcomes, some studies have also shown improvements in inhouse operative teaching, and greater consultant presence in the theatre [11].

## The road ahead is challenging

To date, there has yet to be a universal gold standard as to how to run the ACS system. Although ACS is beginning to show positive results from a systems and workflow point of view; its future is uncertain.

It is therefore imperative to gain insights into ACS systems round the world, to form the basis for learning and comparison. This will ultimately bring the global community together, and will eventually help to foster the development of a universal gold standard system.

## Declarations

### Declarations

No.
